# Race adjustments in clinical algorithms can help correct for racial disparities in data quality

**DOI:** 10.1073/pnas.2402267121

**Published:** 2024-08-13

**Authors:** Anna Zink, Ziad Obermeyer, Emma Pierson

**Affiliations:** ^a^Booth School of Business, University of Chicago, Chicago, IL 60637; ^b^School of Public Health, University of California, Berkeley, CA 94704; ^c^Department of Computer Science, Cornell Tech, New York, NY 10044; ^d^Department of Population Health Sciences, Weill Cornell Medical College, New York, NY 10021

**Keywords:** race adjustments, colorectal cancer, clinical algorithms, family history

## Abstract

Despite ethical and historical arguments for removing race from clinical algorithms, the consequences of removal remain unclear. Here, we highlight a largely undiscussed consideration in this debate: varying data quality of input features across race groups. For example, family history of cancer is an essential predictor in cancer risk prediction algorithms but is less reliably documented for Black participants and may therefore be less predictive of cancer outcomes. Using data from the Southern Community Cohort Study, we assessed whether race adjustments could allow risk prediction models to capture varying data quality by race, focusing on colorectal cancer risk prediction. We analyzed 77,836 adults with no history of colorectal cancer at baseline. The predictive value of self-reported family history was greater for White participants than for Black participants. We compared two cancer risk prediction algorithms—a race-blind algorithm which included standard colorectal cancer risk factors but not race, and a race-adjusted algorithm which additionally included race. Relative to the race-blind algorithm, the race-adjusted algorithm improved predictive performance, as measured by goodness of fit in a likelihood ratio test (*P*-value: <0.001) and area under the receiving operating characteristic curve among Black participants (*P*-value: 0.006). Because the race-blind algorithm underpredicted risk for Black participants, the race-adjusted algorithm increased the fraction of Black participants among the predicted high-risk group, potentially increasing access to screening. More broadly, this study shows that race adjustments may be beneficial when the data quality of key predictors in clinical algorithms differs by race group.

## Significance

This study assessed the impact of race adjustment when the data quality of a key predictor in a colorectal cancer risk prediction algorithm (reported family history of colorectal cancer) varied by race. We found that adjusting for race improved predictive performance and increased the fraction of Black participants among the predicted high-risk group. This study highlights an important and underdiscussed consideration in the debate on race adjustment: When the data quality of input features varies by race, as frequently occurs in clinical settings, algorithms may benefit from race adjustments.

The medical community is locked in a consequential debate over the use of race adjustments in clinical algorithms. Race adjustments incorporate patient race as an input to the algorithm and are used in numerous algorithms across clinical domains (1). The reevaluation of race adjustments is vital and long overdue: Some race adjustments rely on dubious data, exacerbate health disparities, stem from racially biased beliefs, or have been misinterpreted as biological differences between race groups when they are often a result of social determinants of health (1–3). At the same time, the consequences of removing race from clinical algorithms remain unclear, and researchers have called for caution and careful study before doing so (4, 5).

Here, we study an important and largely undiscussed consideration in the race adjustment debate: varying data quality across race groups. Differences in medical data quality by race group, and their consequences for health equity, occur frequently and have been documented in diverse domains (6). When input variables to predictive algorithms are less reliably recorded for some race groups, these variables will tend to have less predictive power for those groups (7–9). Predictive algorithms without race adjustments may fail to capture this, relying too heavily on the unreliable input features for race groups with worse data quality; in contrast, adjusting for race allows clinical algorithms to model differences in the prognostic value of key clinical inputs by race group.

As an example, consider the use of family health history data in cancer risk prediction. Family history of cancer is a known risk factor for many cancers, often resulting in earlier or more frequent screening (10, 11). But recorded family history data, often collected during medical interviews with a clinician, varies in quality across race groups. For example, a number of studies have found that family history of cancer is better known and documented in White patients (12, 13). These racial disparities in data quality mean that the predictive value of recorded family history could vary across race groups. In particular, the absence of recorded family history may be less reassuring in non-White patients, who may be incorrectly recorded as having no family history either because the clinician did not ask, or the patient did not know (14). A race-blind risk prediction would fail to account for this, producing inappropriately low predicted risks for non-White patients without recorded family history; in contrast, race adjustments could model how the prognostic value of recorded family history varies by race and improve prediction.

Using data from the Southern Community Cohort Study (SCCS) (15), established to study cancer disparities, we tested the impact of race adjustment on colorectal cancer prediction when the predictive value of family history of colorectal cancer varied by race group. We created two screening algorithms that modeled 10-y colorectal cancer risk as a function of age, sex, family history, screening history, and lifestyle habits (the same set of controls used by the NIH Colorectal Cancer tool; see *SI Appendix* for a list of variables). The baseline algorithm was race-blind, while the race-adjusted algorithm added Black race both as a main effect and interacted with family history. We compared the two algorithms on several measures of predictive performance including Area Under the Receiving Operating Characteristic Curve (AUC) and calibration. Finally, we assessed how adjusting for race would change the proportion of Black participants flagged as high-risk for colorectal cancer.

## Results

Our sample included 77,836 adults (aged 40 to 74) with no history of colorectal cancer at baseline (Table 1). We first compared the descriptive statistics of self-reported family history (Table 1) for self-reported Black (69.1%) versus White (30.9%) participants. Black participants were more likely than White participants to report unknown family history [Logistic Regression odds ratio (OR): 1.69, 95% CI: 1.58 to 1.81, *P*-value: <0.001], and less likely to report known positive family history (OR: 0.68, 95% CI: 0.64 to 0.72, *P*-value: <0.001) even though Black participants had higher cancer rates. This suggests that self-reported family history might be less reliably recorded for Black participants.

**Table 1.** Sample summary

Variable	Black participants	White participants	All participants
Female (%)	58.4	61.1	59.3
Enrollment age (%)			
40 to 49	48.6	37.7	45.2
50 to 59	35.2	36.2	35.5
60 to 69	13.5	21.9	16.1
70 to 74	2.7	4.1	3.1
Race (%)			
Black	100.0	0	69.1
White	0	100.0	30.9
Family history of colorectal cancer (%)			
Yes	5.9	8.4	6.7
Don't know	7.2	4.4	6.3
Colorectal cancer, 10-y (%)	1.5	1.3	1.4
Colorectal cancer, ever (%)	2.0	1.6	1.9
Mortality (%)	25.3	27.1	25.9
Number of participants	53,805	24,031	77,836

**Fig. 1.**
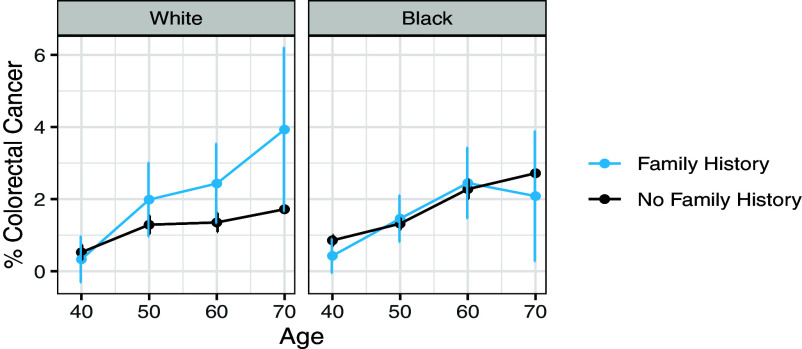
Ten-year colorectal cancer rates by age, family history, and race. Family history was predictive of cancer risk for White participants, but not Black participants.

Based on this, we then compared the prognostic value of self-reported family history (Fig. 1) for Black versus White participants. When we ran separate logistic regressions for Black versus White participants controlling for age and family history (Table 2), we found that family history was strongly predictive of cancer risk for White participants (OR: 1.74, 95% CI: 1.25 to 2.38, *P*-value: 0.001) but not for Black participants (OR: 0.98, 95% CI: 0.72 to 1.29, *P*-value: 0.887).

**Table 2.** Odds ratio (95% CI) for logistic regression predicting 10-y colorectal cancer

Variables	(1) Black participants	(2) White participants	(3) Race-blind algorithm	(4) Race-adjusted algorithm
All data				
Family history	0.979 (0.724 to 1.293)	1.743** (1.246 to 2.383)	1.255* (1.006 to 1.548)	1.802*** (1.285 to 2.467)
Black				1.376*** (1.192 to 1.593)
Family history × Black**^†^**				0.564* (0.366 to 0.873)
Age controls	Y	Y	Y	Y
Full NIH controls			Y	Y

*P* value: <0.001 “***” <0.01 “**” <0.05 “*” < 0.1 “.”.

^†^Family History × Black is the coefficient on the interaction between family history and an indicator for Black race.

Then we compared the race-blind algorithm to the race-adjusted algorithm (Table 2). Both algorithms contained a set of controls based on the NIH Colorectal Cancer tool; the race-adjusted algorithm additionally included race as a main effect and as an interaction with reported family history (16). In the race-blind algorithm, the odds ratio for family history was 1.26 (95% CI: 1.01 to 1.55; *P*-value: 0.038), indicating that participants who reported known family history had 1.26× higher odds of 10-y colorectal cancer than participants who did not. In the race-adjusted algorithm, we found that reported family history was more predictive in White participants than in Black participants: White participants had 1.80× higher odds (95% CI: 1.29 to 2.47; *P*-value: <0.001) of colorectal cancer risk if they reported known family history, whereas Black participants had only 1.02× higher odds (95% CI: 0.75 to 1.34; *P*-value: 0.912). The interaction term between race and family history was 0.56 (95% CI: 0.37 to 0.87; *P*-value: 0.010), indicating that reported family history was considerably less predictive in Black participants than in White participants. Furthermore, the main race effect odds ratio was 1.38 (95% CI: 1.19 to 1.59; *P*-value: <0.001) indicating that, among participants who did not report known family history, Black participants had 1.38× higher odds than White participants of developing colorectal cancer. These higher odds are consistent with prior work reporting higher colorectal cancer rates among Black patients, and likely stem from multiple factors beyond knowledge of family history including disparities in screening, access to care, and environmental factors (17, 18).

The race-adjusted algorithm improved several measures of predictive performance when compared to the race-blind algorithm. First, the race-adjusted algorithm significantly improved goodness of fit (likelihood ratio test, *P*-value: <0.001). Second, on a held-out test set, the race-adjusted algorithm produced a small improvement in AUC among Black participants (0.611 versus 0.608, *P*-value: 0.006, DeLong’s method), and in the overall cohort (0.613 versus 0.606, *P*-value: 0.057), though the increase in AUC among the overall cohort was only statistically significant at the *P* < 0.10 level. The AUC for White participants remained approximately constant (0.613 when adjusting for race versus 0.612 without, *P*-value: 0.586). Finally, as illustrated in *SI Appendix*, Fig. S1, the race-blind algorithm underpredicted risks for Black participants and overpredicted risks for White participants, while the race-adjusted algorithm was better-calibrated for both groups.

**Fig. 2.**
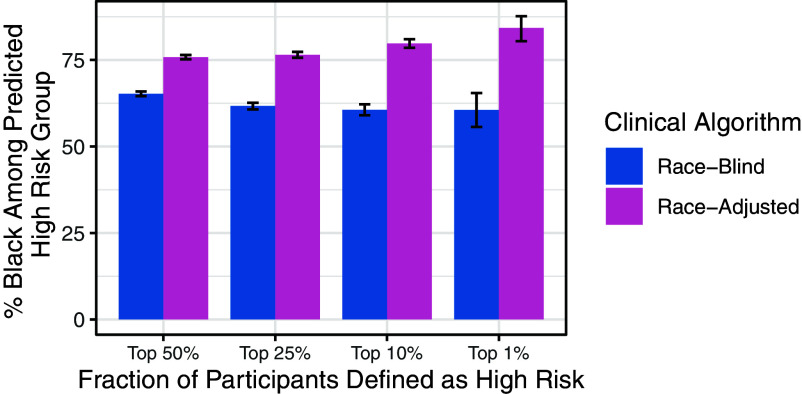
% Black among predicted high risk group, by high risk percentiles. The race-adjusted algorithm included more Black participants among the predicted high-risk group than the race-blind algorithm.

Previous work has raised concerns that race adjustments could increase health disparities by, for example, moving Black patients to lower risk categories and thereby reducing access to screening or other preventive services (1). However, in our setting, we found the opposite effect: The race-adjusted algorithm included a larger share of Black participants among the predicted high-risk group (Fig. 2). With the race-adjusted algorithm, 74.4% of participants flagged in the top 50% of predicted risk were Black compared to 66.1% with the race-blind algorithm (*P*-value: <0.001). Similar results held across all high-risk cutoffs (top quartile, top decile, and top percentile). This is consistent with the fact that the race-blind algorithm underpredicted risks for Black participants, while the race-adjusted algorithm was better calibrated.

Beyond racially disparate underreporting of family history, several other statistical effects might contribute to the results we observed. One contributor might be omitted variable bias, from race-correlated, risk-relevant variables not included in the risk score; to address this, we verified our results were robust to including additional controls, including standard clinical and demographic covariates like age, sex, and lifestyle habits, as well as social determinants of health including education, household income, and health insurance status (*SI Appendix*, Table S2). Second, multiple types of misreporting of family history, not just underreporting, might attenuate the predictive power of family history; to assess this, we verified our results remained robust under alternate ways of encoding family history (*SI Appendix*, Tables S4 and S5). Finally, underreporting of cancer outcomes might affect the results; to address this, we verified our results remained robust under alternate ways of defining the cancer outcome (*SI Appendix*, Table S1). Collectively, these sensitivity analyses suggest that, while other statistical effects may contribute to the results reported above, racially disparate underreporting of family history is an important and robust contributor to the results.

## Discussion

Identifying individuals at high risk for colorectal cancer is an important component of prevention and screening practices in the United States, where colorectal cancer remains the third leading cause of cancer-related death (19). In 2021, the United States Preventive Services Taskforce changed the recommended age for colorectal screening from 50 to 45 in the hopes of increasing screening rates to counteract early-onset cancer, among Black men in particular (20), consistent with findings that colorectal cancer risk varies by race (4). Our analysis found that removing race from colorectal screening predictors could reduce the number of Black patients recommended for screening, which would work against efforts to reduce disparities in colorectal cancer screening and outcomes.

The structural injustices which pervade healthcare and public health mean that critical inputs to medical algorithms—like family history—are more likely to be missing or misrecorded for some race groups (12, 13). Our study illustrated how race adjustments can allow predictive algorithms to model varying data quality across race groups, a frequent phenomenon in health data (6). We focused on disparities in data quality for family history of colorectal cancer. However, analogous disparities in family history data quality may impact risk prediction for other conditions where family history is a risk factor, including breast cancer, cardiovascular disease, and diabetes (21). More broadly, family history is only one of many important risk factors where data quality may vary across race groups. For example, a patient’s own medical history, collected during medical visits and recorded in health care claims or electronic health records, can underreport conditions among those with limited access to care (22, 23).

Future work should examine the implications of varying data quality for race adjustments in other clinical settings, given the pervasive and well-documented differences in clinical data quality by race group. Although we documented a setting where race adjustments improved the clinical risk prediction without increasing health disparities, in other settings, race adjustments may not improve predictive performance or may perpetuate health disparities. Past work has shown that race adjustments may not improve predictive performance if they lead to overfitting (24), or the algorithm is trained to predict a biased label (25, 26). Finally, while adjustment for race may allow algorithms to model deficiencies in medical data as it currently exists, we need to look beyond that to what medical data could be, and must be, if we are to achieve health equity for all patients (27).

## Materials and Methods

### Data.

Our data come from the SCCS established in 2001 to study cancer disparities as well as other health conditions in the southeastern United States (15). SCCS enrollment began in 2002 and continued for 8 y (until 2009). Participants were primarily recruited from community health centers in the following twelve states: Alabama, Arkansas, Florida, Georgia, Kentucky, Louisiana, Mississippi, North Carolina, South Carolina, Tennessee, Virginia, and West Virginia. Data were collected from surveys administered at the time of enrollment and several follow-up periods. Data from the baseline survey were collected either through a self-administered survey or an in-person computer-assisted interview. Follow-up surveys were done by telephone or self-administered: Approximately 68% of participants completed the follow-up surveys. State cancer registry data were linked to participants when possible.

The primary outcome was whether the participant developed colorectal cancer in the 10 y following enrollment. This variable was measured using the follow-up survey, cancer registry data and National Death Index reports of malignant neoplasms of the colon, rectum, and anus. We included all recorded cases of colorectal cancer from any of these three sources.

Family history of cancer was collected for participants’ birth mother, birth father, full sisters, and full brothers (see *SI Appendix*, *Supplemental Appendix* for the SCCS survey codebook). For each family member, respondents could select “yes,” “no,” or “don’t know” for whether the person had cancer. Respondents who indicated that any of these family members had cancer then selected which type of cancer they had. We defined a participant as having a known family history of colorectal cancer if they indicated that one of their family members had colorectal cancer, consistent with previous work (28). For the main analysis, we compared participants with a known family history of cancer to participants who did not have a known family history of cancer (grouping the “don’t know” and “no family history” respondents together in the latter category). In a sensitivity analysis, we considered the effects of two alternate ways of coding family history: 1) analyzing family history as a three-level categorical variable with “don’t know” as a separate category and 2) grouping the “don’t know” group with the “yes” group as opposed to with the “no” group.

All covariates were measured using data collected in the baseline survey. We defined race groups based on the participants’ description of their race or ethnicity at baseline. Participants had six options to choose from (White, Black/African-American, Hispanic/Latino, Asian or Pacific Islander, American Indian or Alaska Native, and Other racial or ethnic group) and could mark all that apply. We defined Black participants as any participants who described themselves as Black/African-American. We defined White participants as any participants who described themselves as White only. More than 95% of the sample identified as either Black or White only, so we only included participants in these two groups for the analysis, following previous work (25).

### Analysis.

We examined the prognostic value of family history by race group by plotting 10-y colorectal cancer rates by age, since age is an important risk factor for colorectal cancer that affects screening recommendations (29). We also ran separate logistic regressions for Black versus White participants in which we predicted 10-y colorectal cancer incidence given age and family history and reported the odds ratio on the family history coefficient for each regression with 95% CI estimated using profile likelihood methods.

Then, we created two screening algorithms that modeled 10-y colorectal cancer risk as a function of age, sex, family history, screening history, and lifestyle habits based on the set of controls used in the NIH Colorectal Cancer tool: participant age at the time of enrollment, an indicator for female, BMI greater than 30, ever had a sigmoidoscopy, ever had a colonoscopy, ever had polyps, the age that the polyp was identified if ever, smoking status (current, former, never), drinking status (<=1 drink per day, >1 drink per day), whether they took Non-steroidal anti-inflammatory drugs (NSAIDs) or Aspirin regularly, whether they did any vigorous activity, and whether they ate vegetables each day (16). One algorithm was race-blind (i.e., did not include race as a predictive feature), whereas the race-adjusted algorithm added an indicator for whether the participant was Black both as a main effect and as an interaction with family history, in addition to the set of controls used by the NIH risk tool (16).

We compared predictive performance of the race-blind and race-adjusted algorithms using two measures. First, we performed a likelihood ratio test to compare goodness of fit in the race-adjusted versus race-blind algorithm. Second, we compared the two algorithms in terms of overall and race-specific AUC, a standard measure of predictive performance, on a holdout test set comprising 50% of the dataset (30). We tested for statistically significant improvements in AUC using DeLong’s algorithm (31).

To assess how the race-adjusted algorithm might impact colorectal cancer screening decisions, we compared the assignment of participants to high-risk strata under the race-blind and race-adjusted algorithms, since participants assigned to high-risk strata are more likely to be screened. We defined predicted high-risk participants as those in the top k% percentile of predicted risk (where k = 50, 25, 10, 5, and 1), and looked at the share of Black participants among the predicted high-risk group. Uncertainty estimates were calculated by bootstrapping the test set and reporting CI across 5,000 bootstrap iterations.

We also performed a set of checks to ensure that reported family history remained more predictive for White participants under different outcome definitions, model choices, and definitions of family history. Please see *SI Appendix* for information on these additional analyses.

## Supplementary Material

Appendix 01 (PDF)

## Data Availability

The data cannot be shared as per the Data Use Agreement, though access to the dataset we used may be obtained through request to the SCCS. Please refer to the following web address for more information on accessing the SCCS data: https://www.southerncommunitystudy.org/research-opportunities.html (32).
