# Supplementary material for: Race adjustments in clinical algorithms can help correct for racial disparities in data quality
Source: Proc Natl Acad Sci U S A. 2024 Aug 13;121(34):e2402267121. doi: 10.1073/pnas.2402267121 (PMC11348319; doi:10.1073/pnas.2402267121)
Supplement: Supplementary file 1 — Appendix 01 (PDF) [file pnas.2402267121.sapp.pdf]

Supporting Information

for

"Race adjustments in clinical algorithms
can help correct for racial disparities in data quality"

Additional Analyses

We also performed a set of checks to ensure that reported family history remained more predictive for White participants under different outcome definitions, model choices, and definitions of family history.

First, we checked that the interaction term between family history and race remained significant under two additional outcome definitions to check that the results were not sensitive to the source of reported data: (1) colorectal cancer cases reported in the follow-up surveys, and (2) colorectal cancer cases found in the state registry data (Table S1). (Most diagnosed cases [approx. 80%] were reported in either the follow-up surveys or the state registry data so we did not separate out national death index cases.) The results remained similar – in particular, family history remained less predictive among Black participants – under these alternate outcome definitions. Note that 31.7% of the sample didn't complete follow-up surveys; and follow-up completion was higher among White participants (74.5%) compared to Black participants (65.6%). We do not know the rates of missingness in the cancer registry data. This raises the question of how underreporting of colorectal cancer among Black participants, relative to White participants, might affect our results. This underreporting bias would likely lead us to *underestimate* the magnitude of effects. Specifically, if underreporting is more likely to affect Black participants, and results in underreporting of both family history of colorectal cancer and colorectal cancer, reported colorectal cancer rates for Black participants without reported family history would be biased downward, reducing the magnitude of the effects we observe. Put another way, the effects we measure would be even larger in a world without this bias, so we do not think underreporting bias explains our results.

Then, we checked to see if our results changed if we included factors related to social determinants of health (SDOH) (Table S2). These factors may help explain some of the loss of predictive power that we see in family history for Black participants, potentially reducing the need for race adjustments. We re-ran the race-adjusted algorithm, including three additional SDOH factors (education status, household income, and insurance coverage) as main effects in one specification, and interacted with family history in a second specification. The inclusion of these additional factors did not change our results: even with their

inclusion we still found similar estimates for the main race term as well as its interaction with family history.

Next, we repeated our examination of the relationship between family history and colorectal cancer using a Cox proportional hazards model, a common choice for modeling time to medical events (Table S3). For our analysis, the time to event was the diagnosis year minus the enrollment year for participants with a diagnosis of colorectal cancer and the censoring year minus the enrollment year for those without. The censoring year was the year of death (if applicable) or 2016 (the most recent year in which all states submitted cancer registry data), whichever occurred first. The results were consistent under this alternative model specification.

Finally, we confirmed that our results were robust to altering the definition of family history (Tables S4-S5). First, rather than grouping the participants with unknown family history with the "no family history" group, we grouped them with the "known family history" group. This might help address the mismeasurement of family history for Black participants if many of those with unknown family history did in fact have a family member with colorectal cancer. We also re-ran the analysis with family history as a categorical variable with three different categories: No, Don't Know, and Yes. The results were robust to these various definitions.

All analyses were run in R version 4.2.1.

Supporting Information References

1. Abd ElHafeez S, D'Arrigo G, Leonardis D, Fusaro M, Tripepi G, Roumeliotis S. Methods to Analyze Time-to-Event Data: The Cox Regression Analysis. *Oxid Med Cell Longev*. 2021;2021:e1302811. doi:10.1155/2021/1302811

2. Robin X, Turck N, Hainard A, et al. pROC: an open-source package for R and S+ to analyze and compare ROC curves. *BMC Bioinformatics*. 2011;12(1):77. doi:10.1186/1471-2105-12-77

Table S1. Odds Ratio (95% Confidence Intervals) for Logistic Regression Predicting 10-Year Colorectal Cancer

Variables	(1) Black Participants	(2) White Participants	(3) Race-Blind Algorithm	(4) Race-Adjusted Algorithm
All Data				
Family History	0.979 (0.724 to 1.293)	1.743** (1.246 to 2.383)	1.255* (1.006 to 1.548)	1.802*** (1.285 to 2.467)
Black				1.376*** (1.192 to 1.593)
Family History × Black†				0.564* (0.366 to 0.873)
Follow-Up Survey Data‡				
Family History	0.939 (0.604 to 1.389)	2.068** (1.376 to 3.007)	1.317 . (0.983 to 1.729)	2.022*** (1.343 to 2.949)
Black				1.205 . (0.997 to 1.463)
Family History × Black†				0.458** (0.257 to 0.807)
Cancer Registry Data				
Family History	1.068 (0.751 to 1.473)	1.626* (1.065 to 2.390)	1.328* (1.017 to 1.705)	1.794** (1.173 to 2.643)
Black				1.370*** (1.151 to 1.638)
Family History × Black†				0.633 (0.375 to 1.077)
Age Controls	Y	Y	Y	Y
Full NIH Controls			Y	Y

P Value: <0.001 '***' <0.01 '**' <0.05 '*' < 0.1 '.'

†Family History × Black is the coefficient on the interaction between family history and an indicator for Black race.

‡Excludes 31.7% of the sample that didn't complete follow-up surveys. 34.4% of Black participants did not complete follow-up surveys and 25.5% of white participants did not complete follow-up surveys.

Note: The coefficient on the interaction term remains similar across all three specifications (though the confidence interval is wider in the final specification and thus not statistically significant) indicating that, across our robustness checks, family history was consistently less predictive for Black than White participants.

Table S2. Odds Ratio (95% Confidence Intervals) for Logistic Regression Predicting 10-Year Colorectal Cancer, including SDOH factors (Education, Household Income, and Insurance Status)

Variables	(1) No SDOH	(3) SDOH Main Effects	(4) SDOH interaction
Family History	1.802*** (1.285 to 2.467)	1.801*** (1.285 to 2.465)	1.834** (1.211 to 2.702)
Black	1.376*** (1.192 to 1.593)	1.334*** (1.155 to 1.546)	1.332*** (1.153 to 1.544)
Family History × Black†	0.564* (0.366 to 0.873)	0.568* (0.368 to 0.880)	0.570* (0.367 to 0.888)
Age Controls		Y	Y
Full NIH Controls		Y	Y

P Value: <0.001 '***' <0.01 '**' <0.05 '*' < 0.1 '.'
†Family History × Black is the coefficient on the interaction between family history and an indicator for Black race.

Regression includes full set of NIH controls. SDOH variables included indicators for participants with 11 or fewer years of education, participants who reported household income <$15,000, and participants who indicated they were uninsured. Thresholds were chosen to balance people in each group. Results were robust to using more granular categories of household income and education.

Table S3. Hazard Ratios (95% Confidence Intervals) for Cox Proportional Hazard Model Predicting 10-Year Colorectal Cancer

Variables	(1) Black Participants	(2) White Participants	(3) Race-Blind Algorithm	(4) Race-Adjusted Algorithm
Family History	0.995 (0.765 to 1.296)	1.722*** (1.275 to 2.326)	1.254* (1.029 to 1.528)	1.766*** (1.306 to 2.387)
Black				1.304*** (1.139 to 1.493)
Family History × Black†				0.583** (0.391 to 0.870)
Age Controls	Y	Y	Y	Y
Full NIH Controls			Y	Y

P Value: <0.001 '***' <0.01 '**' <0.05 '*' < 0.1 '.'

†Family History × Black is the coefficient on the interaction between family history and an indicator for Black race.

Note: The coefficient on the interaction term remains similar using a Cox Proportional Hazard model indicating that, across our robustness checks, family history was consistently less predictive for Black than White participants.

Table S4. Odds Ratio (95% Confidence Intervals) for Logistic Regression Predicting 10-Year Colorectal Cancer using Alternative Family History Definition

Variables	(1) Black Participants	(2) White Participants	(4) Race-Blind Algorithm	(5) Race-Adjusted Algorithm
Family History†	1.100<br>(0.901 to 1.331)	1.596**<br>(1.189 to 2.108)	1.237*<br>(1.050 to 1.449)	1.591**<br>(1.185 to 2.103)
Black				1.372***<br>(1.183 to 1.597)
Family History × Black†				0.695*<br>(0.494 to 0.987)
Age Controls	Y	Y	Y	Y
Full NIH Controls			Y	Y

P Value: <0.001 '***' <0.01 '**' <0.05 '*' < 0.1 '.'

†Family history used here groups participants who don't know whether a family member has had colorectal cancer or not with participants who report a known family history. Family History × Black is the coefficient on the interaction between family history and an indicator for Black race.

Note: The coefficient on the interaction term remains similar when we use an alternative definition of family history indicating that, across our robustness checks, family history was consistently less predictive for Black than White participants.

Table S5. Odds Ratio (95% Confidence Intervals) for Logistic Regression Predicting 10-Year Colorectal Cancer using 3-level Categorical Family History Definition

Variables	(1) Black Participants	(2) White Participants	(3) Race-Blind Algorithm	(4) Race-Adjusted Algorithm
Family History (Don't Know) †	1.187 (0.921 to 1.506)	1.263 (0.728 to 2.033)	1.201 (0.955 to 1.490)	1.184 (0.682 to 1.190)
Family History (Yes) †	0.995 (0.735 to 1.315)	1.766** (1.260 to 2.419)	1.273* (1.019 to 1.571)	1.818*** (1.295 to 2.494)
Black				1.371*** (1.182 to 1.595)
Family History (Don't Know) × Black†				0.985 (0.573 to 1.792)
Family History (Yes) × Black†				0.566* (0.366 to 0.878)
Age Controls	Y	Y	Y	Y
Full NIH Controls			Y	Y

P Value: <0.001 '***' <0.01 '**' <0.05 '*' < 0.1 '.'

†Family history used here has three categories (No, Don't Know, and Yes). No family history is the reference group. Family History × Black is the coefficient on the interaction between the family history category and an indicator for Black race.

Note: The coefficient on the interaction term Family History (Yes) × Black remains similar when we use an alternative definition of family history indicating that, across our robustness checks, family history was consistently less predictive for Black than White participants.

Table S6. AUC in Race-Blind versus Race-Adjusted Algorithm in Holdout Sample

Participants	Race-Blind AUC	Race-Adjusted AUC	Increase in AUC	P Value for 2-sided test
All	0.606	0.613	0.007	0.057 .
Black	0.608	0.611	0.003	0.006**
White	0.612	0.613	0.002	0.586

P Value: <0.001 '***' <0.01. '**' <0.05 '*' < 0.1 '.'

Note: P-values were calculated using the function *roc.test* in the R package pROC[2], which compared paired ROC curves using DeLong's algorithm.

Figure S1. Actual vs. Predicted 10-Year Colorectal Cancer Risk

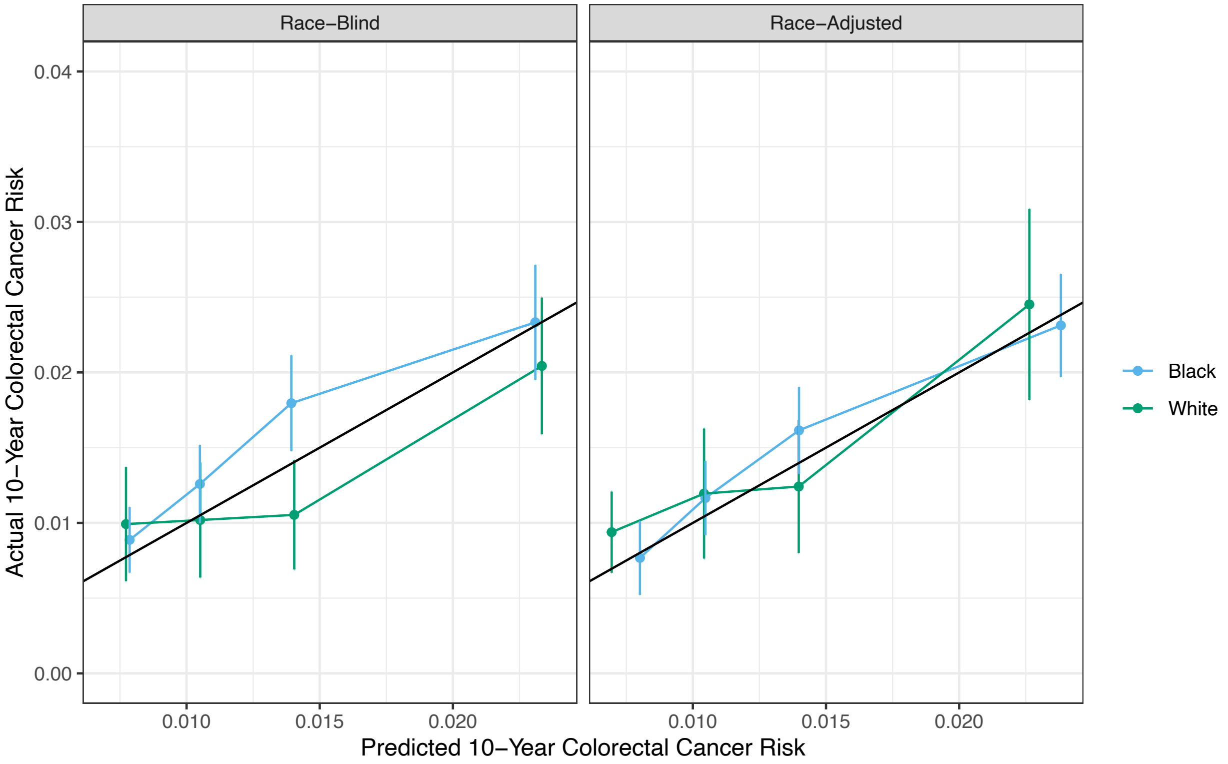

Note: The race-adjusted algorithm (right) yielded better-calibrated estimates than the race-blind algorithm (left). The horizontal axis plots predicted risk, and the vertical axis plots true risk, for quartiles of the test set. Perfectly calibrated estimates, where predicted risk and true risk are equivalent, would lie along the black diagonal line. The race-blind algorithm yielded predictions which are too low for Black participants; in contrast, the race-adjusted algorithm mitigated this issue (decrease in expected calibration error for Black participants: 0.00121, 95% CI: 0.0012-0.0028 from 5,000 bootstrap iterations).

Supplemental Appendix: SCCS Variables and Codebook Questions

**SCCS Variable Name**	**SCCS Question and Coding**
Demographics Variables (Baseline Survey)	
Enrollment_Age	Age at interview (years)<br>Numeric value (integer) from 40 to 79
Sex	What is the participant's gender?<br>M = Male<br>F= Female
RaceAnalysis	Analysis variable to use for race<br>1 = White (only)<br>2 = Black/African-American (only)<br>3 = Hispanic/Latino (any)<br>4= Asian or Pacific Islander (only)<br>5 = American Indian or Alaska Native (only)<br>6 = Other racial or ethnic group (only)<br>7 = Mixed race (excluding Hispanic/Latino)<br>8888 = Refuse<br>9999 = Don't know
RaceWhite	Did the participant report "White" as all or part of his/her racial or ethnic background?<br>0 = No<br>1 = Yes<br>8888 = Refuse<br>9999 = Don't know
RaceBlack	Did the participant report "Black/African American" as all or part of his/her racial or<br>ethnic background?<br>0 = No<br>1 = Yes<br>8888 = Refuse<br>9999 = Don't know
Other Variables (Baseline Survey)	
InsuranceCoverage	Are you covered by any type of health insurance including private insurance, Medicare or Medicaid?<br>0 = No<br>1 = Yes<br>8888 = Refuse<br>9999 = Don't know
HHIncome	Which of the following describes your total household income last year?<br>1 = Less than $15,000<br>2 = At least $15,000 but less than $25,000 3 = At least $25,000 but less than $50,000<br>4 = At least $50,000 but less than $100,000<br>5 = $100,000 or more<br>8888 = Refuse<br>9999 = Don't know

Education	What is the highest grade or level of education you have completed?<br><br>1 = Less than 9 years<br>2 = 9-11 years<br>3 = 12 years, completed high school, or GED<br>4 = Vocational, technical, or business training<br>5 = Some college or junior college<br>6 = Graduated from college<br>7 = Graduate school (up to and including Master's degree)<br>8 = Graduate school beyond a Master's degree (include doctors, dentists, lawyers, PhDs)<br>8888 = Refuse<br>9999 = Don't know
Family History Variables (Baseline Survey)	
Mo ColonCancer	Did participant report that his/her birth mother had colon cancer?<br>0 = No<br>1 = Yes<br>7777 = Not applicable<br>8888 = Refuse<br>9999 = Don't know
Mo ColorectalCancer	Did participant report that his/her birth mother had colorectal cancer?<br>0 = No<br>1 = Yes<br>7777 = Not applicable<br>8888 = Refuse<br>9999 = Don't know
Mo RectalCancer	Did participant report that his/her birth mother had rectal cancer?<br>0 = No<br>1 = Yes<br>7777 = Not applicable<br>8888 = Refuse<br>9999 = Don't know
Fa_ColonCancer	Did participant report that his/her birth father had colon cancer?<br>0 = No<br>1 = Yes<br>7777 = Not applicable<br>8888 = Refuse<br>9999 = Don't know
Fa_ColorectalCancer	Did participant report that his/her birth father had colorectal cancer?<br>0 = No<br>1 = Yes<br>7777 = Not applicable<br>8888 = Refuse<br>9999 = Don't know
Fa_RectalCancer	Did participant report that his/her birth father had rectal cancer?<br>0 = No<br>1 = Yes<br>7777 = Not applicable<br>8888 = Refuse<br>9999 = Don't know
Br_ColonCancer	Did participant report that any brothers had colon cancer?<br>0 = No<br>1 = Yes

	7777 = Not applicable<br>8888 = Refuse<br>9999 = Don't know
Br_ColorectalCancer	Did participant report that any brothers had colorectal cancer?<br>0 = No<br>1 = Yes<br>7777 = Not applicable<br>8888 = Refuse<br>9999 = Don't know
Br_RectalCancer	Did participant report that any brothers had rectal cancer?<br>0 = No<br>1 = Yes<br>7777 = Not applicable<br>8888 = Refuse<br>9999 = Don't know
Si_ColonCancer	Did participant report that any sisters had colon cancer?<br>0 = No<br>1 = Yes<br>7777 = Not applicable<br>8888 = Refuse<br>9999 = Don't know
Si_ColorectalCancer	Did participant report that any sisters had colorectal cancer?<br>0 = No<br>1 = Yes<br>7777 = Not applicable<br>8888 = Refuse<br>9999 = Don't know
Si_RectalCancer	Did participant report that any sisters had rectal cancer?<br>0 = No<br>1 = Yes<br>7777 = Not applicable<br>8888 = Refuse<br>9999 = Don't know
Covariates (Baseline Survey)	
BMICat	Body Mass Index categories<br>1 = <185<br>7 = 185 -<25.0<br>3=25.0-<30.0<br>4 = 30.0 -< 35.0<br>5= 250_< 40 0<br>6 = 40+
Sigmoidoscopy_Ever	Have you ever had a sigmoidoscopy?<br>0 = No<br>1 = Yes<br>8888 = Refuse<br>9999 = Don't know
Colonoscopy_Ever	Have you ever had a colonoscopy?<br>0 = No<br>1= Yes<br>8888 = Refuse<br>9999 = Don't know
Polyps	Has a doctor ever told you that you have had polyps in your colon or rectum?<br>0= No<br>1 = Yes

	8888 = Refuse<br>9999 = Don't know
Polyps_AFD	What was your age at your first diagnosis of polyps in your colon or rectum?<br>Numeric value (years) from 18 to 79<br>7777 = Not applicable<br>8888 = Refuse<br>9999 = Don't know
SmokingStatus	Cigarette smoking status<br>1=Current<br>2=Former<br>3=Never<br><br>7777 = Not applicable
AlcoholPerDay	Number of drinks per day. The sum of LightBeer_Freq*Beer_Quant, RegBeer_Freq*Beer_Quant, WhiteWine_Freq*WhiteWine_Quant, RedWine_Freq*RedWine_Quant, and Liquor_Freq*Liquor_Quant, where each frequency was converted to a decimal representing number of times per day.
RxNSAIDS	In the past year, have you taken the following medication regularly? The prescription drugs Celebrex, Vioxx, or Bextra<br><br>0 = No<br>1 = Yes<br>7777 = Not applicable<br>8888 = Refuse<br>9999 = Don't know
Aspirin	In the past year, have you taken the following medication regularly? Regular aspirin (such as Anacin, Bayer, Bufferin, Excedrin, etc.)<br><br>0 = No<br>1 = Yes<br>8888 = Refuse<br>9999 = Don't know
VigActivity_hrs	Total vigorous activity hours
VegPerDay > .5	How many times per day did you typically eat: vegetables? Numeric value from 0.5 to 15<br><br>0.5 = Less than once per day<br>8888 = Refuse<br>9999 = Don't know
Colorectal Cancer (Baseline Survey)	
ColorectalCancer	Did participant report having had colorectal cancer?<br>0 = No<br>1 = Yes<br>7777 = Not applicable<br>8888 = Refuse<br>9999 = Don't know
ColonCancer	Did participant report having had colon cancer?<br>0 = No<br>1 = Yes<br>7777 = Not applicable<br>8888 = Refuse<br>9999 = Don't know

RectalCancer	Did participant report having had rectal cancer?<br>0 = No<br>1 = Yes<br>7777 = Not applicable<br>8888 = Refuse<br>9999 = Don't know
Colorectal Cancer (Follow-up Surveys)	
ColorectalCancerF1-F3	Has the participant reported a diagnosis of colorectal cancer?<br>0 = No<br>1 = Yes<br>7777 = Not applicable<br>8888 = Refuse<br>9999 = Don't know
ColonCancerF1-F3	Has the participant reported a diagnosis of colon cancer?<br>0 = No<br>1 = Yes<br>7777 = Not applicable<br>8888 = Refuse<br>9999 = Don't know
RectalCancerF1-F3	Has the participant reported a diagnosis of rectal cancer?<br>0 = No<br>1 = Yes<br>7777 = Not applicable<br>8888 = Refuse<br>9999 = Don't know
Colorectal Cancer (Cancer Registry Data)	
Colorectal_Cancer	Flag indicating cancer was a colorectal cancer. 1 ICD-O-3 Primary Site of C18.0-C18.9, C19.9, C20.9; excluding the following histologies: 9590-9989, 9050-9055, 9140+; invasive behavior (behavior_icdo3 = 3)
Colorectal Cancer (Mortality Data)	
NDI_Recode	023 Malignant neoplasms of colon, rectum, and anus

A link all questionnaire codebooks can be found at the following link: https://www.southerncommunitystudy.org/codebooks-and-documentation.html.
